# Artificial intelligence-assisted psychological intervention mechanisms for university students in the context of new media technologies: an analysis based on data from the national institute of mental health

**DOI:** 10.3389/fpsyg.2025.1619818

**Published:** 2025-09-03

**Authors:** Na Hao, Meng Chen, Ning Zhao, Jun Zhang, Fanyu Zheng

**Affiliations:** ^1^School of Art & Design, Guangzhou College of Commerce, Guangzhou, China; ^2^Faculty of Humanities and Arts, Macau University of Science and Technology, Macau, China; ^3^School of Art and Design, Guangzhou University, Guangzhou, China; ^4^School of Public Administration, Guangzhou University, Guangzhou, China

**Keywords:** university student mental health, artificial intelligence, intervention strategies, personalized intervention, new media

## Abstract

**Introduction:**

With the pervasive influence of new media, mental health issues among college students have become increasingly prominent, manifested in high rates of emotional disorders, delayed interventions, uneven resource allocation, and limited attention to individual differences. This study aims to assess the mental health status of college students and explore the potential of artificial intelligence (AI) technologies in supporting psychological interventions.

**Methods:**

Publicly available data from the National Institute of Mental Health (NIMH) were analyzed using descriptive statistical methods. Key variables included depression, anxiety, social support, quality of life, and technology usage. Based on these findings, a personalized AI intervention framework was developed, integrating psychological assessments, social support levels, and technology usage patterns.

**Results:**

The analysis revealed that the average depression score among the student population was 7.5 (*SD* = 4.2), and the average anxiety score was 6.8 (*SD* = 3.9), highlighting the widespread prevalence of emotional issues. A significant correlation was also identified between technology usage frequency and negative psychological indicators.

**Discussion:**

The study demonstrates the novelty of applying AI models to psychological interventions by leveraging intelligent perception, real-time feedback, and dynamic adjustment mechanisms. This personalized and data-driven approach enhances the efficiency and precision of mental health support. These findings provide both a theoretical foundation and a practical pathway for developing innovative mental health support systems in higher education.

## 1 Introduction

With the rapid advancement of new media technologies, tools such as social media, mobile applications, and virtual reality have profoundly influenced individuals' social behaviors, learning processes, and mental health (Lattie et al., 2019). University students, a demographic heavily reliant on new media technologies, are increasingly exposed to complex mental health challenges. The high-intensity flow of information and mounting social pressures have significantly contributed to the rising prevalence of mental health issues, including anxiety and depression, within this group (Yu and Li, 2022). Traditional mental health intervention approaches, which often lack personalization and real-time responsiveness, have proven insufficient to address these evolving challenges (Xu, 2024).

The integration of artificial intelligence (AI) into psychological health interventions offers innovative solutions to these issues. Techniques such as natural language processing (NLP), emotion recognition, and personalized recommendation systems enable AI to detect mental health concerns in real time and provide targeted, adaptive intervention strategies (Fu and Qiao, 2023). When combined with new media technologies, AI has the potential to deliver tailored and immediate mental health support, especially for university students who are deeply embedded in digital environments (Fulmer et al., 2018). Despite its potential, the application of AI in mental health care is not without challenges. Concerns surrounding data privacy, the sustainability of intervention outcomes, and the adaptability of AI models to diverse user scenarios remain significant obstacles. Addressing these challenges is essential for the broader adoption and effectiveness of AI-driven psychological health interventions.

The mental health of university students significantly influences their academic performance, social adaptability, future career development, and broader societal stability. Consequently, developing effective psychological intervention mechanisms tailored to the unique needs of university students has become an urgent priority. Traditional psychological interventions, such as face-to-face counseling, have proven effective in specific situations but face critical challenges, including limited resources, high costs, and a lack of personalized support. These limitations render conventional methods insufficient to address the growing mental health needs of the student population (Chen J. et al., 2024). In this context, integrating AI with new media technologies offers transformative potential by enabling large-scale coverage and delivering personalized, real-time mental health support (Li et al., 2023).

University mental health intervention systems still predominantly rely on traditional face-to-face counseling and linear assessment models, which often suffer from delayed feedback, limited personalization, and insufficient responsiveness to dynamic psychological changes. Some studies have explored using AI for emotion recognition and risk screening. However, fully integrating AI technologies into university mental health frameworks remains rare. Leveraging big data to provide personalized, real-time, and dynamically adaptive interventions is still limited. As a result, the diverse and evolving mental health needs of students are largely unmet.

To address these gaps, this study introduces an AI-driven personalized intervention framework that integrates psychological assessment data, social support measures, and technology usage patterns. Drawing on large-scale datasets from the National Institute of Mental Health (NIMH), the framework identifies key factors associated with depression and anxiety while employing intelligent algorithms for dynamic monitoring of mental states and precise, individualized strategy recommendations. By enhancing personalization, continuity, and responsiveness, the proposed approach advances beyond traditional intervention models, bridging critical technological and practical gaps in the digital transformation of university mental health services.

## 2 Literature review

### 2.1 Current state of university students' mental health

In recent years, mental health issues among university students have garnered increasing attention from both academia and society. The prevalence of psychological challenges such as anxiety, depression, and stress has been rising globally. According to (Asher BlackDeer et al. 2023), over 30% of university students worldwide experience varying levels of anxiety or depression during their academic journey. Similarly, mental health concerns among Chinese university students are significant. (Zhi 2023) reported that more than 20% of Chinese university students exhibit notable psychological issues, with academic pressure, societal expectations, and employment uncertainties serving as primary contributing factors. Additionally, (Acosta-Gonzaga 2023) highlighted that mental health problems in this demographic not only impede academic performance but also carry long-term negative implications for career development and social adaptability.

The mental health challenges faced by university students also exhibit distinct characteristics compared to the general adult population. These issues are often intricately tied to academic performance, campus social relationships, and future career planning. For instance, (Mofatteh 2021) identified academic pressure as a leading driver of anxiety and depression among university students. This psychological strain is frequently amplified by the pervasive influence of social media, which can exacerbate feelings of stress and inadequacy. Understanding the current state of university students' mental health and identifying the specific factors contributing to their challenges are crucial for developing effective intervention strategies. A nuanced approach that addresses the unique pressures faced by this population is essential to mitigate the adverse effects on their academic and personal lives while promoting long-term wellbeing.

### 2.2 Application of AI in mental health

The rapid advancement of AI technology in recent years has facilitated its integration into mental health assessment and intervention. AI's powerful data processing capabilities, particularly in emotion recognition and NLP, have enabled effective analysis of mental health data and the identification of potential psychological issues. (Thakkar et al. 2024) reviewed the field and emphasized significant progress in AI applications for emotion recognition, mental health assessment, and intervention design. Leveraging NLP techniques, AI can automatically detect emotional characteristics such as depression and anxiety from textual or speech data, offering valuable diagnostic tools for mental health professionals.

For instance, (Roy et al. 2020) demonstrated AI's potential to evaluate mental health through social media data. By applying sentiment analysis techniques, they extracted emotional trends from tweets to predict risks of depression. Additionally, AI can construct predictive models to forecast an individual's future mental health risks and generate personalized intervention recommendations tailored to user characteristics (Ramírez-Cifuentes et al., 2020). While these technologies show significant promise, research focusing specifically on university students remains limited. (Budiarto et al. 2024) introduced an e-learning assessment system grounded in the principles of Outcome-Based Education (OBE), which intelligently generated personalized learning recommendations and intervention strategies based on learners' performance and competency levels. This data-driven assessment and recommendation mechanism parallels the design of personalized mental health intervention programs, as both prioritize precision support and dynamic adaptability. These similarities underscore the broad potential of AI technologies in advancing individualized development and health management. Given the unique psychological challenges faced by this demographic, this study aims to address the gap by investigating the targeted application of AI in mental health interventions for university students. Such an approach has the potential to enhance the precision and effectiveness of interventions, catering to the distinct needs of this population.

### 2.3 Impact of new media technologies on university students' mental health

New media technologies, particularly the pervasive use of social media, have profoundly transformed the social behavior and information acquisition patterns of university students. While these technologies offer abundant learning resources and social opportunities, they have also introduced significant mental health challenges, including cyberbullying, information overload, and identity crises (Sweeney et al., 2024). Research by (Jasso Medrano and Lopez Rosales 2018) revealed a strong correlation between excessive social media use and symptoms of anxiety and depression among university students. This negative impact is particularly evident among students with low levels of social support or self-efficacy. Additionally, the “comparison culture” promoted by social media often fosters dissatisfaction with oneself, further exacerbating mental health problems.

Conversely, new media technologies also hold potential as effective tools for mental health interventions. Chen Q. et al. (2024) found that utilizing new media platforms to disseminate mental health knowledge and deliver psychological support services significantly improved the accessibility of mental health care. Building on these insights, this study aims to integrate new media technologies with AI to develop personalized, real-time mental health intervention strategies, offering targeted solutions to alleviate psychological issues among university students. (Saldívar-Almorejo et al. 2024) demonstrated in their study on e-learning in STEM education that effectively managing the challenges of technology integration and implementing well-designed strategies markedly improved graduates' employment outcomes. These findings provided valuable insights into how new media technologies positively influenced university students' educational experiences and psychological adaptation. They also emphasized the need to balance opportunities and potential challenges when designing mental health interventions, ensuring that educational progress and psychological support were advanced in tandem.

### 2.4 Mental health research based on large-scale data

The analysis of large-scale datasets has become increasingly integral to mental health research, enabling investigations that extend beyond individual cases to encompass population-level insights. Datasets provided by the NIMH serve as a foundational resource for exploring mental health trends and associated factors. For example, (Kammer-Kerwick et al. 2024) analyzed NIMH data to examine the prevalence of common mental health disorders among United States adults, uncovering correlations with variables such as age, gender, and race. These datasets not only facilitate the identification and description of mental health issues but also support the prediction of individual mental health risks using techniques like regression analysis and machine learning.

In the domain of university students' mental health, large-scale datasets have been utilized to assess and predict psychological challenges. (Fu and Bin 2022) used extensive survey data to analyze mental health trends among university students and to model the influence of various contributing factors. However, such studies often rely on traditional statistical methods, limiting their ability to capture the nuanced interactions within the data. This study seeks to address this limitation by integrating large-scale datasets with advanced AI techniques. By combining the predictive capabilities of AI with the breadth of population-level data, the study aims to provide deeper insights into mental health prediction and intervention strategies tailored to the unique needs of university students. For example, (Al-Ali et al. 2024) developed a student performance prediction model by analyzing sociological and academic factors using machine learning methods. Their work highlights the critical role of multidimensional data in forecasting student behavior and academic outcomes. Such research provides valuable methodological support for integrating large-scale data and AI technologies to deeply explore students' mental health status. This insight informs the present study's approach, which leverages AI algorithms applied to datasets from the NIMH to dynamically optimize personalized psychological intervention strategies.

### 2.5 Theoretical foundations of psychological interventions

The effectiveness of psychological interventions for college students largely hinges on the scientific rigor and applicability of their theoretical foundations. Cognitive Behavioral Theory (CBT) is extensively employed in contemporary intervention models. CBT posits that emotional distress arises from maladaptive cognitive patterns, and that modifying these irrational cognitions can lead to improvements in emotional wellbeing and behavior (Maher and King, 2023). Among college students, CBT has been effectively implemented through group counseling and online interventions addressing anxiety, depression, social phobia, and related issues, demonstrating strong practical utility and empirical validation. Mindfulness-Based Cognitive Therapy (MBCT), a relatively recent intervention approach, has gained increasing prominence within university mental health programs. MBCT focuses on cultivating non-judgmental awareness of present-moment experiences, thereby enhancing students' capacity for emotional regulation and stress management.

Self-Determination Theory (SDT) offers a motivational framework for understanding psychological health, identifying autonomy, competence, and relatedness as fundamental components that promote wellbeing (Gagné et al., 2022). Recently, SDT has been integrated into AI-driven interactive systems to refine personalized interventions, utilizing adaptive feedback mechanisms that bolster user engagement and self-efficacy. In data-driven intervention research, the Health Belief Model is commonly applied to explain students' acceptance or resistance toward psychological intervention recommendations. Core constructs of this model include perceived threat, perceived benefits, perceived barriers, and cues to action, providing a theoretical basis for designing interventions in new media environments. Collectively, these theoretical frameworks provide comprehensive support for developing effective intervention strategies and mechanisms. As AI technologies advance, the convergence of theoretical insight and algorithmic innovation is anticipated to further enhance the precision, personalization, and intelligence of mental health interventions targeting college students.

### 2.6 Literature summary

Current literature has extensively documented the significant mental health challenges faced by college students, alongside the promising applications of AI in emotion recognition, mental health assessment, and personalized intervention. Concurrently, the pervasive adoption of new media technologies has deeply influenced students' psychological wellbeing and created new opportunities for the digital transformation of mental health services. Furthermore, empirical research utilizing large-scale datasets has progressively enhanced macro-level understanding of mental health issues and facilitated the development of predictive models. Despite these advances, several gaps persist in the existing literature. Most studies concentrate on single-variable analyses or rely on traditional statistical approaches, lacking comprehensive intervention frameworks that integrate multidimensional psychological indicators, social support, and technology usage behaviors. Moreover, the deployment of AI technologies for personalized and dynamic mental health interventions within university settings remains limited, with insufficient empirical validation and algorithmic refinement. To address these shortcomings, the present study proposes an innovative AI-driven personalized psychological intervention model that integrates psychological scale data, social support assessments, and technology usage patterns. This model is designed to enable real-time dynamic monitoring and precise intervention of college students' mental health states, thereby enhancing both the effectiveness and responsiveness of interventions. It fills a critical gap in the development of intelligent, comprehensive mental health intervention systems and offers both theoretical and practical contributions toward the digital advancement of mental health services in higher education.

## 3 Research methodology

### 3.1 Data source

The data used in this study is derived from the mental health survey dataset provided by the NIMH. This publicly available dataset was collected by the United States government as part of its long-term tracking and analysis of mental health conditions across various populations, offering broad applicability and significant scientific value. The dataset includes multidimensional information on mental health, such as emotional disorders (e.g., anxiety and depression), utilization of mental health services, interactions with the social environment, and socio-economic background variables. The dataset underwent rigorous de-identification during the collection process, ensuring that no personally identifiable information was included. This approach complies with ethical standards and data usage requirements, safeguarding the privacy and rights of participants.

Given that the focus of this study is on university students, the first step in data processing involved filtering the dataset to include individuals aged 18 to 25. This age range encompasses both undergraduate and graduate students, making it pertinent for examining the mental health characteristics of university students, particularly in relation to academic pressure, environmental changes, and increased independence. Additionally, the selection criteria ensured that the dataset exclusively included individuals currently enrolled in higher education, further refining the focus of the study. After screening, the study included a valid sample of 484 participants, with a missing data rate of 2.3%. Detailed descriptive statistics are provided to ensure transparency and data integrity. The NIMH dataset primarily reflects the mental health status of college students in the United States; therefore, the generalizability of findings should be limited to student populations with similar cultural backgrounds. Following data screening, several key variables closely related to college students' mental health were extracted, including:

1. Anxiety level: assessed using the Generalized Anxiety Disorder-7 (GAD-7) scale, a 7-item self-report questionnaire measuring the frequency of anxiety symptoms over the past 2 weeks. Scores range from 0 to 21, with higher scores indicating greater anxiety severity. The scale demonstrated good reliability in this dataset, with a Cronbach's α of 0.89.
2. Depressive symptoms: evaluated by the Patient Health Questionnaire-9 (PHQ-9), which includes 9 items assessing depressive symptoms over the past 2 weeks. Scores range from 0 to 27, with higher scores indicating more severe depression. The internal consistency reliability of the scale was α = 0.86.
3. Stress level: measured using the Perceived Stress Scale-10 (PSS-10), a 10-item instrument reflecting subjective evaluations of stress experienced recently. Scores range from 0 to 40, with higher scores indicating greater perceived stress. The reliability coefficient α was 0.84.
4. Use of mental health services: recorded whether participants had sought psychological counseling or treatment and included frequency categories such as “never,” “occasionally,” or “frequently.”
5. Social behavior: captured social interaction status, including the size of social circles, frequency of offline social activities, and usage of online social platforms, reflecting social engagement and support levels.
6. Academic performance: constructed from self-reported grades, perceived academic pressure, and study habits to analyze the relationship between academic burden and mental health. Combined with other psychological variables, this aids in improving model prediction accuracy.

**Figure 1:**
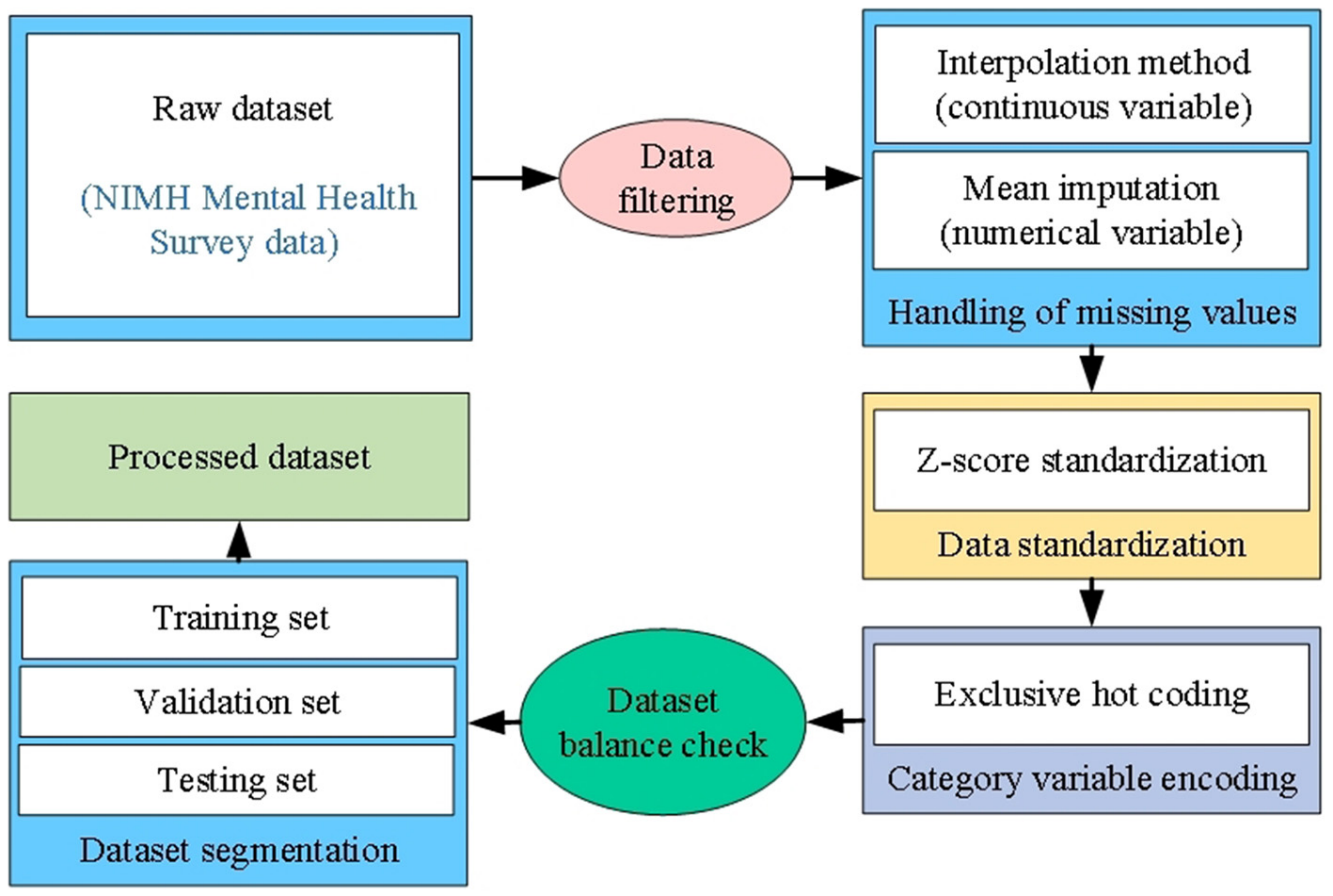
Data processing workflow[[Inline Image]].

The next step in data processing involves cleaning and preprocessing the selected variables. The specific procedures are illustrated in Figure 1.

For the filtered dataset, interpolation and mean imputation methods were employed to address the small number of missing values, ensuring that incomplete data did not negatively impact the model's performance. For numerical variables (e.g., anxiety and depression scores), standardization was applied to ensure balanced weighting across different variables in subsequent analyses and model training. The Z-score standardization method was used, transforming numerical variables (e.g., anxiety and depression scores) into a standard normal distribution with a mean of 0 and a standard deviation of 1. This approach not only accelerates the model's convergence but also improves the model's sensitivity to varying data types. The formula for Z-score standardization is as Equation 1:


(1) $$\begin{array}{l} Z=\frac{X-\mu }{\sigma } \end{array}$$

In Equation 1, *Z* signifies the standardized value, *X* denotes the original value, μ is the mean of the variable, and σ represents the standard deviation of the variable.

The mental health dataset also includes categorical variables, such as the use of mental health services, gender, and race. To prepare these variables for input into the AI model, one-hot encoding was applied to convert categorical variables into numerical form. For instance, the “Use of Mental Health Services” variable, with two possible values (Yes/No), was transformed into two binary vectors: Use of Services (Yes) → [1, 0]; No Services Used (No) → [0, 1]. The processed dataset ensures data integrity and consistency, providing high-quality input for subsequent model training. Through careful selection and preprocessing steps, the data reflects the mental health status of university students with precision, establishing a solid foundation for further analysis.

### 3.2 AI model design

Based on a clear understanding of the research data, this study designed and implemented a reinforcement learning–based model to assess the application of AI technology in mental health interventions for university students. The model aims to dynamically optimize intervention strategies and deliver personalized psychological support tailored to students' varying mental health conditions. The core architecture of the model consists of three key components: mental health status prediction, intervention strategy selection, and intervention optimization based on reinforcement learning. The model utilizes reinforcement learning, where the student's mental health status is treated as the state of the environment, interventions are regarded as the model's actions, and the effectiveness of these interventions is quantified through a reward function. The primary goal is to enable the system to learn the optimal strategy, allowing it to select the most suitable intervention for different mental health conditions and, ultimately, assist students in improving their psychological wellbeing.

**Figure 2:**
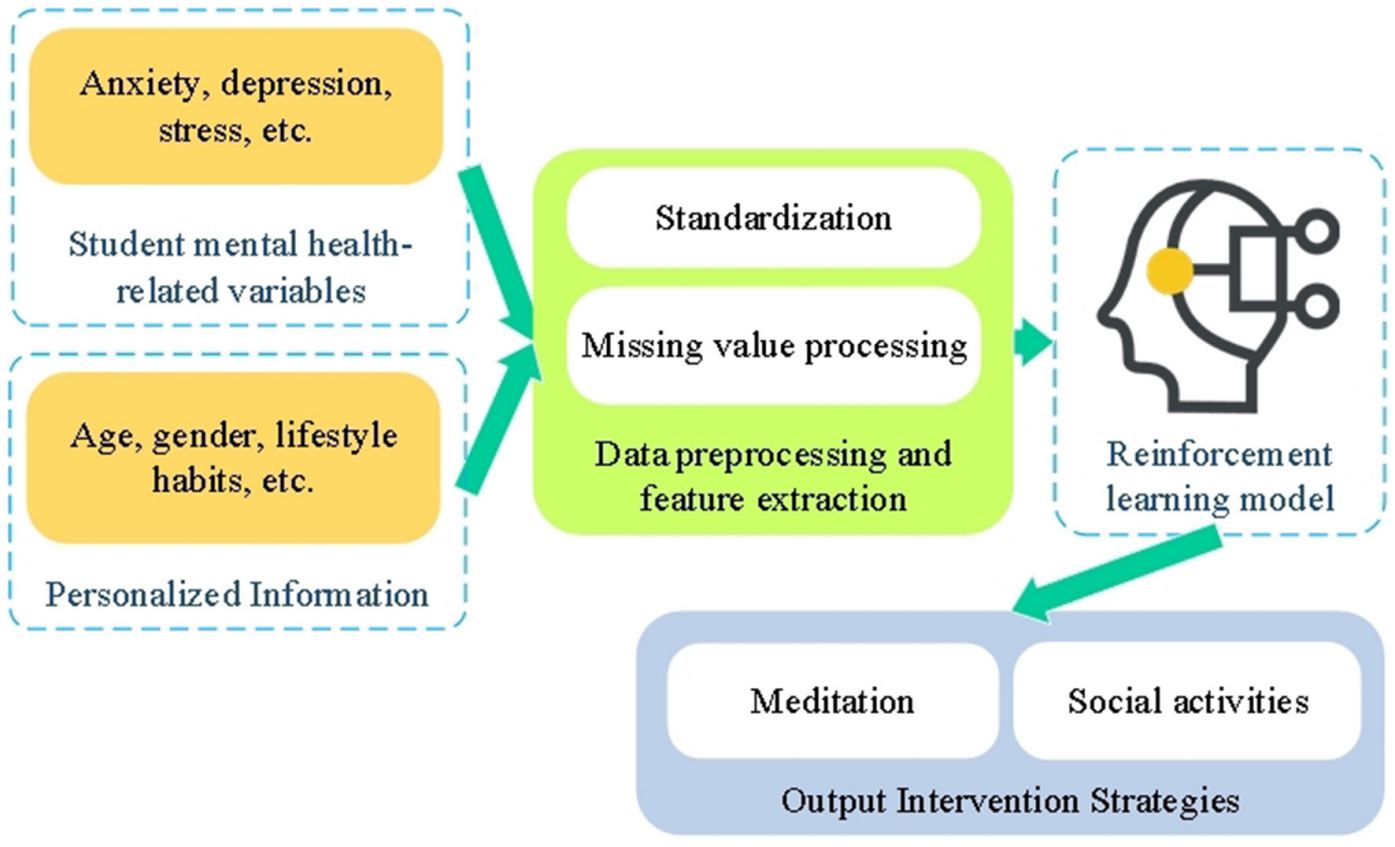
Reinforcement learning-based mental health intervention model.

Inputs to the model include quantified data on mental health-related variables such as anxiety, depression, and stress symptoms, drawn from the NIMH dataset, along with personalized student information (e.g., age, gender, and lifestyle habits). The output is the selection of an intervention strategy tailored to provide the most appropriate mental health support for each individual student. A schematic representation of the model is shown in Figure 2.

The reinforcement learning model, illustrated in Figure 2, is designed to dynamically optimize intervention strategies for improving students' mental health. Key elements of the model include the state space (S), action space (A), and reward function (R), which collectively define the framework for personalized intervention. The state space represents the mental health status of students, encompassing dimensions such as anxiety, depression, and stress. These variables are derived from the NIMH mental health survey data after undergoing rigorous preprocessing and standardization. The state space is mathematically expressed as Equation 2:


(2) $$\begin{array}{l} S=\left(s_{1},s_{2},s_{3},\ldots s_{n}\right) \end{array}$$

In Equation 2, *s*_1_, *s*_2_, *s*_3_, …*s*_*n*_ are the quantified values of different mental health dimensions (e.g., anxiety, depression, and stress). To accurately capture the dynamic changes in students' mental health over time, this study designed a state transition mechanism within the reinforcement learning model's environment. The indicators defining the environment state evolve at each time step, influenced by the previous state and the intervention actions taken. The state transition function is based on the assumption that the current mental health state and the intervention effects jointly determine the state at the next time step. Mathematically, this is expressed as:


(3) $$\begin{array}{l} s_{t+1}=g\left(s_{t},a_{t}\right)+\epsilon \end{array}$$

where *s*_*t*_ represents the mental health state vector at time *t*, *a*_*t*_ denotes the intervention action applied, *g(*·*)* describes the impact of the intervention on the mental health state, and ϵ is a stochastic noise term modeling natural fluctuations and measurement errors in mental health.

To construct the function g, this study combined longitudinal survey data from the NIMH dataset and established intervention effect patterns from psychological literature. A linear weighted model was used to estimate the intervention effects, ensuring the state transition simulation aligns with real-world mental health change trends. Moreover, the environment dynamics account for latent delay effects, recognizing that some intervention impacts may manifest several time steps after implementation. This is modeled by incorporating a time-delay mechanism to reflect such realistic features.

The action space defines the set of possible intervention strategies available for different mental health states. Building on a discrete action framework, this study extended it by introducing a hybrid action mechanism. This combines discrete actions with continuous parameters (e.g., intervention intensity, duration) at certain intervention points, enhancing the expressiveness and personalization of strategies while maintaining system controllability and computational feasibility. The action space is formalized as:


(4) $$\begin{array}{l} A=\left\{\left(a_{i},\theta_{i}\right)|a_{i}\in A_{d},\theta_{i}\in A_{c}\left(a_{i}\right)\right\} \end{array}$$

(5) $$\begin{array}{l} A_{d}=\left\{a_{1},a_{2},\ldots ,a_{m}\right\} \end{array}$$

$A_{d}$ denotes the discrete set of intervention actions, such as cognitive behavioral therapy, mindfulness training, exercise recommendations, or social activity suggestions. The continuous parameter θ_*i*_ is associated with a specific discrete action *a*_*i*_, representing adjustable factors like intensity, frequency, or duration. The set $A_{c}\left(a_{i}\right)$ defines the continuous parameter space available after choosing action *a*_*i*_. For example, if *a*_2_ corresponds to mindfulness training, θ_2_ might represent the daily practice duration (in minutes). If *a*_3_ is physical exercise, θ_3_ could represent training intensity or weekly frequency.

In Equation 3, *a*_1_, *a*_2_, *a*_3_, …*a*_*m*_ represent various intervention strategies designed to address specific mental health challenges. The reward function is a fundamental component of the reinforcement learning model, designed to quantify the effectiveness of selected intervention strategies in improving mental health outcomes. Its primary purpose is to measure changes in students' mental health status, such as reductions in symptoms of anxiety or depression. Positive rewards are assigned for effective interventions that lead to improvements, while negative rewards are given for ineffective interventions. A set of multidimensional mental health variables is selected, including anxiety, depression, and stress. The reward function is defined as a weighted combination of the changes in these variables, mathematically expressed as Equation (6):


(6) $$\begin{array}{l} R=f\left(\text{Δ}s_{1},\text{Δ}s_{2},\text{Δ}s_{3},\ldots ,\text{Δ}s_{n}\right) \end{array}$$

*s*_*i*_ represents the change in the i-th mental health indicator after the intervention, such as the reduction in anxiety scores or the improvement in depression scores. A positive value indicates an improvement in mental health, for which the model assigns a positive reward, encouraging the selection of that intervention strategy. Conversely, a negative value reflects deterioration, resulting in a negative reward to discourage that strategy. To more accurately reflect the relative importance of different indicators to overall mental health, the reward function incorporates weighted allocations, emphasizing the contributions of more critical psychological variables. This design not only ensures a comprehensive reward signal but also enhances the model's sensitivity to the multidimensional mental health state. During the reinforcement learning process, the Q-learning algorithm was employed. It guides the choice of intervention strategies by continuously updating the Q-values—action-value functions—for state-action pairs. The update rule for the Q-values follows Equation 7:


(7) $$Q(s_{t},a_{t})=Q(s_{t},a_{t})+\alpha (R_{t+1}+\gamma max_{a'}Q(s_{t+1},a')-Q(s_{t},a_{t}))$$

Here, *Q*(*s*_*t*_, *a*_*t*_) represents the Q-value for taking action *a*_*t*_ in state *t*; *R*_*t*+1_ signifies the reward received after taking action *a*_*t*_ in state *s*_*t*_; γ is the discount factor, which determines the weight of future rewards in the current decision; α denotes the learning rate, controlling the magnitude of Q-value updates. Through iterative updates, the model progressively improves its selection of intervention strategies by maximizing long-term cumulative rewards.

**Figure 3:**
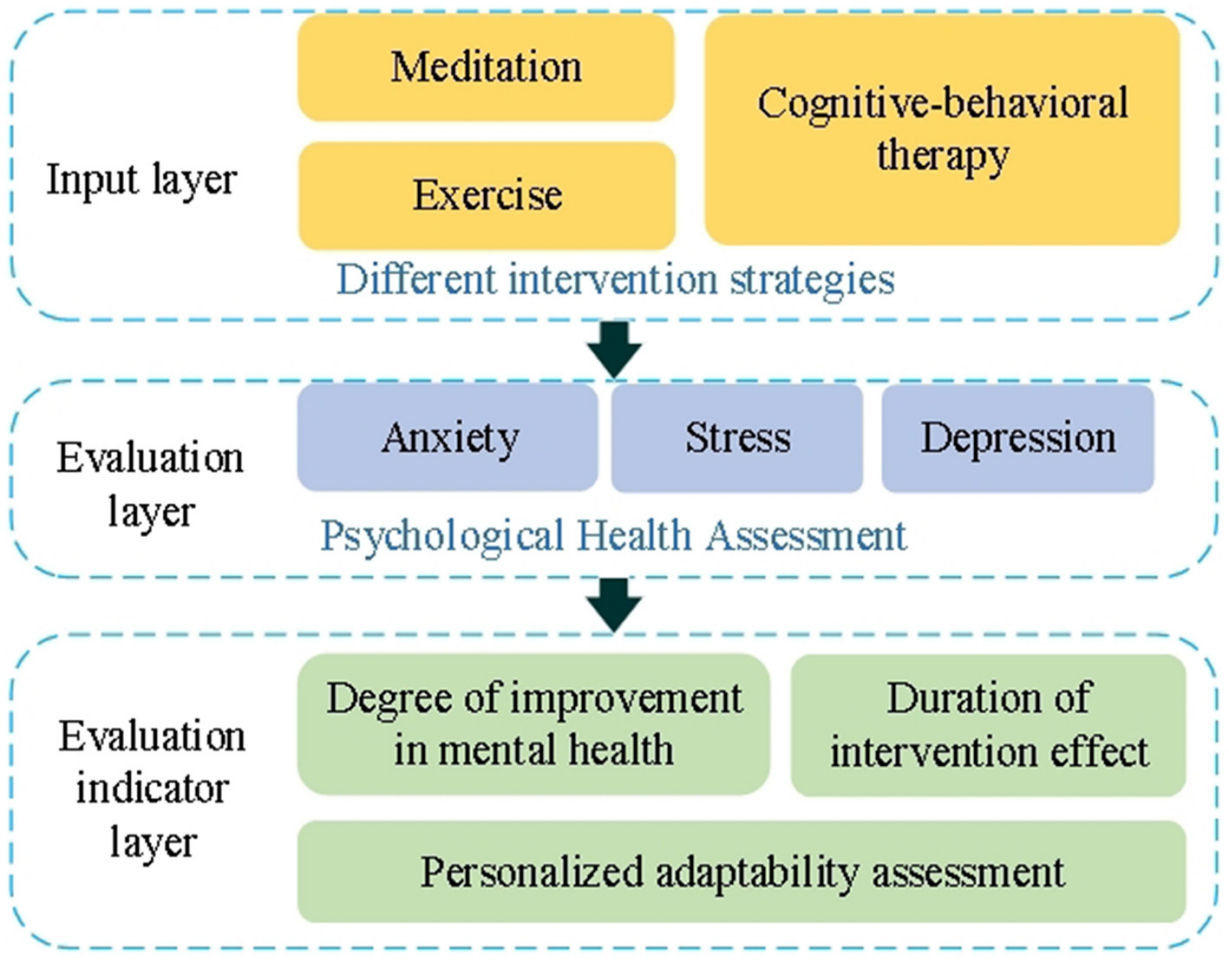
Process of psychological intervention model evaluation.

During the training phase, the model optimizes its intervention strategy through continuous interaction with the environment, which simulates changes in students' mental health status. The training dataset includes variables such as students' mental health status, lifestyle habits, and social interactions. At each training step, the model selects an intervention strategy based on the current state, receives feedback in the form of rewards, and updates the Q-value until convergence is achieved. The evaluation of the model involves comparing the effectiveness of different intervention strategies. Key evaluation indicators include the extent of improvement in students' mental health, the duration of the intervention's effect, and the adaptability of the intervention to individual needs. The process of evaluating the psychological intervention model is illustrated in Figure 3.

To ensure the ethical compliance of the system's recommendations and the scientific rigor of psychological interventions, an Ethical Constraint Layer was incorporated into the reinforcement learning policy. This module functions as a filtering mechanism for policy outputs and embeds a rule base derived from national mental health guidelines and clinical intervention standards. Each candidate action generated by the system is evaluated against these ethical criteria. Actions that potentially induce negative emotions, violate ethical principles of psychological intervention, or pose ethical risks are dynamically filtered out. The module then selects and returns the optimal feasible action that complies with ethical requirements, thereby safeguarding the ethical integrity of the recommendation process.

### 3.3 Experimental design and parameter analysis

To validate the effectiveness and feasibility of the proposed reinforcement learning model in the context of university student mental health interventions, a systematic experimental framework was designed. This framework encompasses hyperparameter configuration, training procedures, and sensitivity analyses, adhering to principles of reproducibility, stability, and convergence.

The model training environment was implemented using Python 3.9 and TensorFlow 2.13, running on an Intel(R) Core(TM) i9 CPU with 32GB RAM and an NVIDIA RTX 3090 GPU. The dataset was preprocessed and partitioned into training (70%), validation (15%), and test sets (15%) via random sampling without replacement to ensure generalizability and objective evaluation. Each training epoch comprised ~10,000 simulated intervention steps, during which the model iteratively learned optimal policies through continuous interaction with the simulated environment.

The state space represents individual students' scores across psychological dimensions such as anxiety, depression, and stress. The action space includes a variety of possible interventions, for example, recommendations for cognitive behavioral therapy, social engagement, and mindfulness training. The reward function assesses the effectiveness of each intervention. Policy optimization was performed using the Q-learning algorithm, with Q-values updated iteratively until convergence. Table 1 summarizes the key hyperparameters and their rationale:

**Table 1:** Key hyperparameter settings[[Inline Image]].

**Hyperparameter**	**Symbol**	**Value**	**Rationale**
Learning rate	α	0.05	Tuned within 0.01–0.1 range to balance stability and convergence speed.
Discount factor	γ	0.8	Balances immediate feedback with long-term intervention benefits.
Exploration rate (Init)	ϵ	0.3 → 0.05 (decay)	Encourages exploration early on, decaying over training to ensure stability.
Reward weighting	–	Adaptive	Dynamically adjusts weights according to changes in psychological metrics.
Max iterations	–	3,000 epochs	Set based on average convergence observed on the validation dataset.

The hyperparameters listed above were chosen based on common practices in mainstream reinforcement learning research. They were also adjusted to fit the specific characteristics of this study's scenario. In particular, psychological state changes tend to be gradual, and intervention strategies require long-term feedback. To evaluate the impact of these key hyperparameters on model performance, a sensitivity analysis was conducted. Three core hyperparameters—learning rate (α), discount factor (γ), and initial exploration rate (ϵ)—were each tested within specified ranges through five experimental combinations. Their effects on intervention effectiveness (measured primarily by the reduction in anxiety scores) and convergence speed were assessed. During training, the average decrease in anxiety and depression scores served as the main performance metric. After each training epoch, the intervention strategy was evaluated on the validation set; if no significant improvement was observed for 20 consecutive epochs (defined as < 0.5% change), the model was considered to have converged.

Given that the NIMH dataset used in this study is cross-sectional and lacks real intervention follow-up data, a simulation experiment based on the reinforcement learning model was designed to evaluate intervention strategies. Initially, the psychological health status data of the screened college students were used as the initial environment states. The model then simulated changes in students' mental health indicators (such as anxiety and depression) under different intervention actions, guided by the optimal policy learned during training. In this simulation, the reinforcement learning model dynamically selected the most appropriate intervention at each step according to the current psychological state, thereby generating intervention pathways and predicting corresponding mental health improvements. To validate the model's effectiveness, the simulation results were compared against random and fixed intervention strategies, focusing on symptom improvement magnitude and convergence stability.

### 3.4 Data analysis and evaluation of influencing factors

Upon completion of model training and verification of performance stability, an in-depth data-level analysis was conducted to identify key factors influencing college students' mental health. This section examines key factors influencing university students' mental health and the effectiveness of AI-assisted psychological intervention mechanisms through data analysis. To assess the impact of these factors on intervention outcomes, multiple variables from the NIMH dataset were selected, including mental health status, social support, quality of life, demographic information, and technology usage patterns. By analyzing these variables, it becomes possible to identify those that significantly affect the success of mental health interventions, offering data-driven insights to inform future strategies.

Mental health status is a central focus of the analysis. Depression and generalized anxiety disorder were chosen as key indicators to measure anxiety and depression levels among university students, as these scales are commonly used in mental health assessments. Regression analysis is employed to evaluate the relationships between the scores of these two conditions and other variables such as quality of life and social support. This approach enables the identification of connections between mental health status and external factors, forming a foundation for the development of more targeted and effective intervention strategies.

In addition to mental health status, the impact of social support and quality of life on university students' mental health was also analyzed. Social support refers to the assistance and care individuals receive from family, friends, and social networks, while quality of life measures an individual's overall satisfaction with their life circumstances. These factors may play a mitigating role in mental health interventions, particularly in alleviating symptoms of anxiety and depression. By analyzing scores on the social support and quality of life scales, key aspects that positively influence mental health can be identified, providing valuable data for the development of personalized intervention strategies.

Demographic information, including age, gender, year of study, and academic major, was also considered in the analysis. These variables are instrumental in assessing the mental health needs of different student groups. For example, students in certain academic years or disciplines may experience heightened academic pressure, potentially leading to more severe mental health challenges. Analyzing the relationships between these demographic variables and mental health scores enables the design of more targeted interventions, ultimately improving the effectiveness of mental health support for various student groups.

The use of technology is another critical factor influencing university students' mental health. The integration of smartphones and social media into daily life has become ubiquitous among university students. Research has indicated that excessive use of these technologies may exacerbate symptoms of anxiety and depression. Therefore, the relationship between the frequency of smartphone use, social media usage, and mental health status was analyzed to explore how these technological factors affect the mental health intervention mechanism. Data analysis revealed potential correlations between technology usage and mental health, offering valuable evidence for the development of technology-assisted mental health interventions. Various statistical methods were employed during the data analysis process, including descriptive statistics and correlation analysis. Pearson correlation analysis was used to examine the linear relationships between mental health status and factors such as social support and quality of life.

## 4 Results and discussion

### 4.1 Descriptive statistics of mental health status

**Figure 4:**
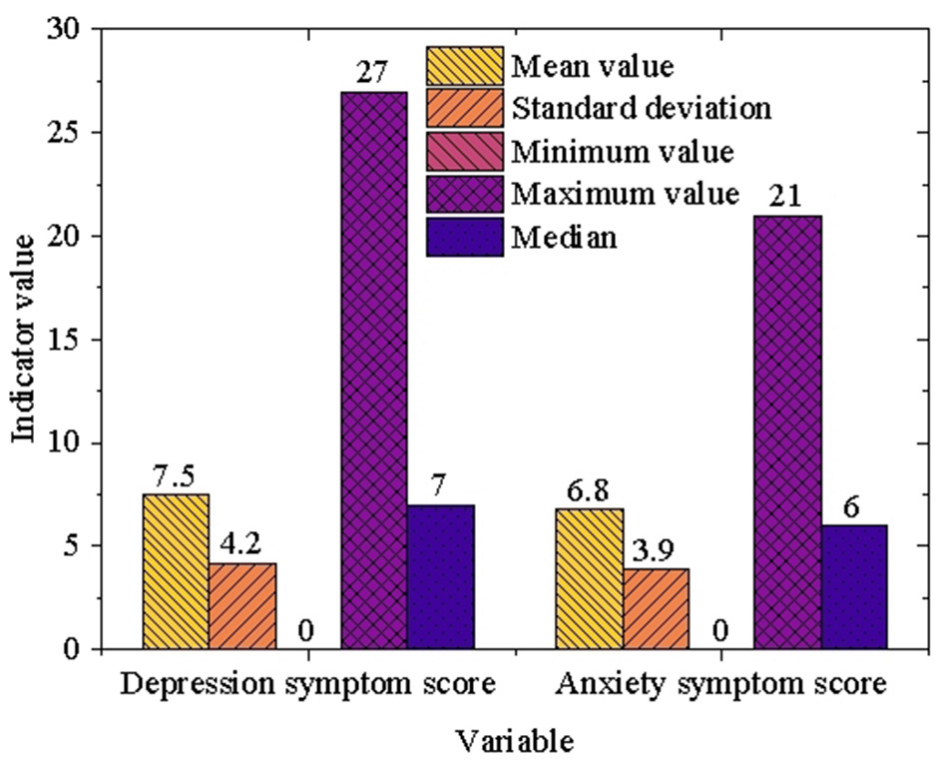
Descriptive statistics of university students' mental health status.

To comprehensively assess the mental health status of university students, the mental health survey data provided by NIMH was utilized, with a focus on depression and anxiety symptoms. Figure 4 presents the descriptive statistics related to these aspects of mental health among university students.

As shown in Figure 4, the average depression score among university students is 7.5, with a standard deviation of 4.2, indicating a broad and diverse range of depression symptoms across the population. The median score is 7, reflecting that most students experience mild to moderate levels of depression rather than severe cases. This result highlights not only the prevalence of depressive symptoms within the student population but also the variability in their intensity. Similarly, the average anxiety score is 6.8, accompanied by a standard deviation of 3.9, which also points to considerable variability in anxiety symptoms among students. The median score of 6 reinforces the observation that the majority of students experience mild to moderate anxiety, with fewer individuals showing either minimal or severe symptoms. These findings underscore the prominence of anxiety as a significant concern among university students, with a wide range of severities observed. A closer comparison of the depression and anxiety scores reveals similar distribution patterns, both characterized by high variability and moderate severity. This suggests that mental health interventions should account for these individual differences to provide more tailored and effective support for students, addressing the unique challenges they face.

### 4.2 Social support and quality of life statistics

**Figure 5:**
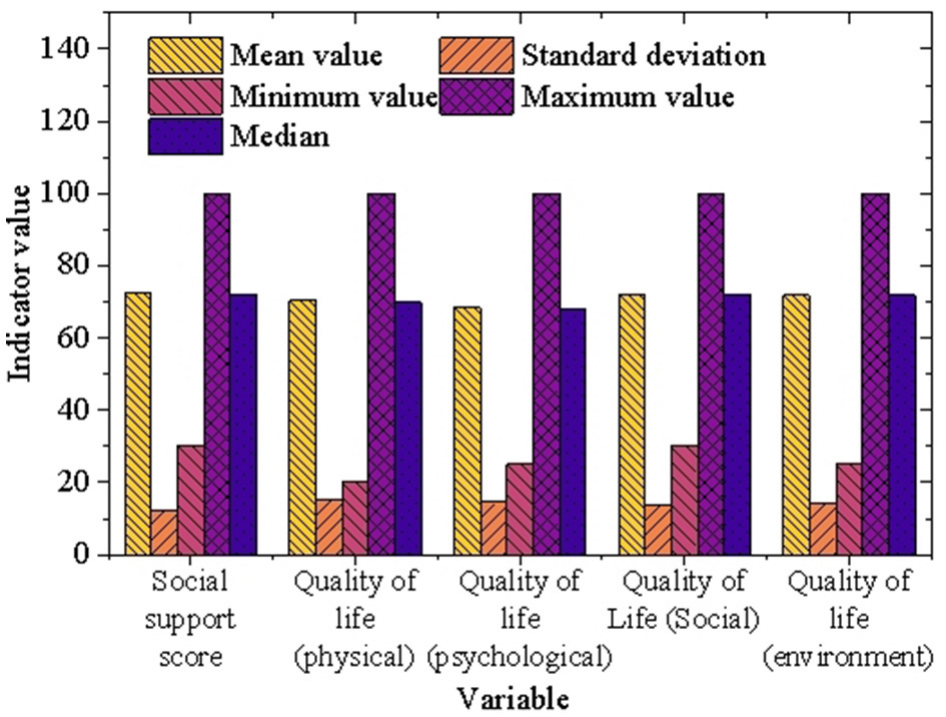
Descriptive statistics of social support and quality of life among university students.

To thoroughly assess university students' social support and quality of life, data from the NIMH mental health survey were utilized, focusing on various dimensions of these factors. Figure 5 presents the descriptive statistics for social support and quality of life among university students.

As shown in Figure 5, the average social support score for university students is 72.3, with a standard deviation of 12.5, indicating that most students receive a relatively high level of social support. The median score of 72 further reinforces this finding, suggesting that university students generally benefit from substantial support systems, including family, friends, and peers, especially when facing psychological stress. This robust social support contributes positively to their mental health and overall wellbeing. In terms of quality of life, university students report a generally favorable overall quality of life, with average scores for each dimension as follows: physical health (70.5), mental health (68.3), social relationships (72.1), and environmental quality (71.8). The standard deviations for these dimensions−15.2, 14.5, 13.8, and 14.1, respectively—indicate that most scores are relatively concentrated, although some students report significantly lower quality of life in specific areas.

Specifically, the average physical health score of 70.5, with a standard deviation of 15.2, suggests that while most students are satisfied with their physical health, a subset experiences notable health concerns. The average mental health score of 68.3, with a standard deviation of 14.5, reflects that although many students generally feel positive about their mental health, a portion struggles with psychological distress. Similarly, the average score for social relationships is 72.1, with a standard deviation of 13.8, highlighting that most students are satisfied with their social interactions and maintain good relationships, though a smaller group faces challenges in this area. Finally, the average environmental quality score of 71.8, with a standard deviation of 14.1, indicates that most students are content with their living environments, despite some expressing dissatisfaction with certain environmental factors.

### 4.3 Statistics on the use of new media technologies

**Figure 6:**
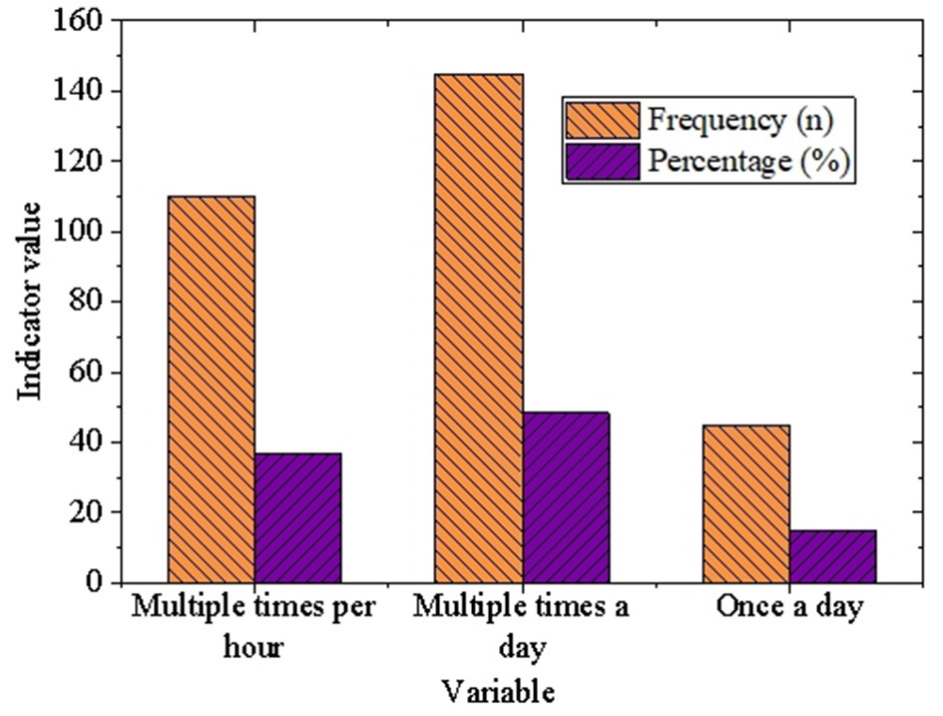
Descriptive statistics of smartphone usage among university students.

To evaluate the technology usage patterns of university students, mental health survey data provided by NIMH were analyzed, with a focus on the frequency of smartphone and social media use. Figure 6 presents the descriptive statistical results for these aspects among college students.

**Figure 7:**
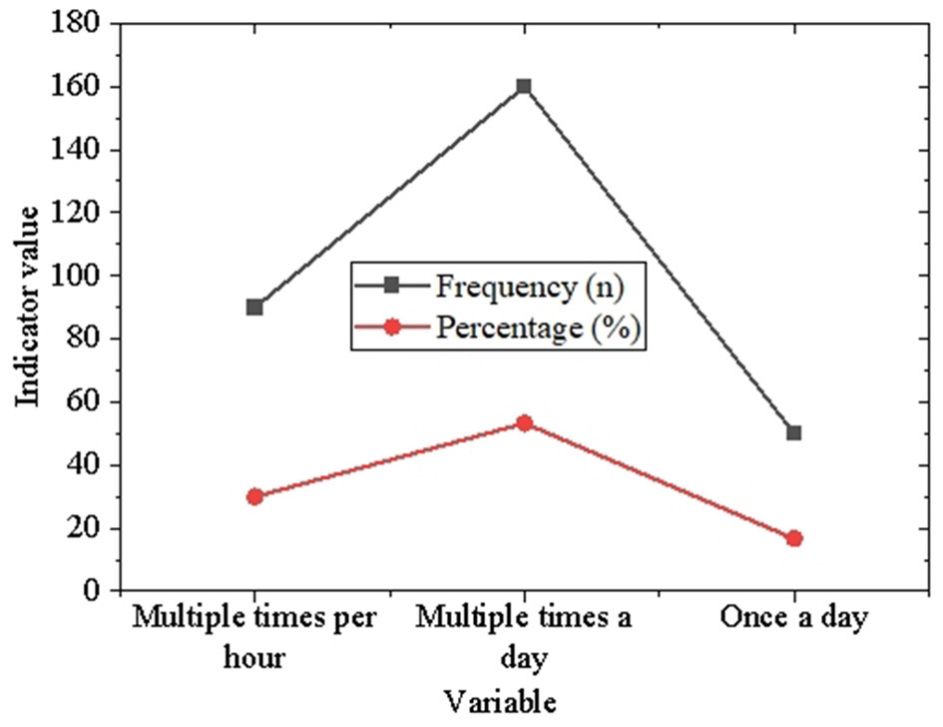
Descriptive statistics of social media usage among university students.

As illustrated in Figures 6, 7, university students exhibit a remarkably high frequency of technology usage. Specifically, in terms of smartphone usage, 48.3% of students report using smartphones multiple times a day, 36.7% use them multiple times per hour, and 15.0% use them once a day. This underscores the central role smartphones play in the daily routines of university students, serving as indispensable tools for communication, information access, and entertainment. Regarding social media usage, 53.3% of students engage with social media multiple times a day, 30.0% do so multiple times per hour, and 16.7% access social media once a day. These findings further highlight the pervasive adoption of social media among university students, showcasing its significance as a platform for both acquiring information and maintaining social connections.

### 4.4 Hyperparameter sensitivity analysis

To evaluate the robustness and adaptability of the proposed AI-based psychological intervention model under different parameter settings, sensitivity experiments were conducted on three key hyperparameters: learning rate (α), discount factor (γ), and maximum training epochs. Keeping other parameters constant, each hyperparameter was varied individually to assess its impact on model intervention effectiveness and convergence performance. For the learning rate α, five experimental groups were set within the range {0.1, 0.3, 0.5, 0.7, 0.9}, with corresponding training and testing results summarized in Table 2.

**Table 2:** Effect of learning rate α on model performance.

**Learning rate α**	**Average convergence epochs**	**Average cumulative reward**	**Anxiety score reduction**	**Depression score reduction**
0.1	2,720	42.8	1.9	2.0
0.3	2,100	51.5	2.5	2.9
0.5	1,750	57.8	2.8	3.3
0.7	1,850	54.3	2.6	3.0
0.9	2,050	48.7	2.2	2.6

As shown in Table 2, a learning rate of 0.5 achieved the best balance between convergence speed and intervention effectiveness. Lower values (e.g., 0.1) led to stable but prolonged training, while higher values (e.g., 0.9) caused noticeable instability and inferior intervention outcomes compared to moderate learning rates. The discount factor γ balances the model's emphasis on immediate rewards vs. long-term intervention benefits. Larger γ values prioritize long-term strategy outcomes. Under fixed conditions (α = 0.5, maximum training epochs = 3,000), model performance was tested across γ values {0.6, 0.7, 0.8, 0.9, 0.99}, with results presented in Table 3. The model performed best at γ = 0.8, effectively capturing both short-term feedback and long-term psychological improvements. This parameter was adopted in subsequent experiments.

**Table 3:** Effect of discount factor γ on model performance.

**Discount factor γ**	**Average convergence epochs**	**Average cumulative reward**	**Anxiety score reduction**	**Depression score reduction**
0.6	1,680	49.5	2.3	2.8
0.7	1,750	53.1	2.6	3.0
0.8	1,800	57.4	2.8	3.4
0.9	1,880	54.0	2.5	3.1
0.99	2,000	50.7	2.2	2.7

Although a maximum number of training epochs was set, an Early Stopping strategy was introduced to terminate training if the cumulative reward showed no significant improvement over 20 consecutive epochs. To evaluate this mechanism, training was conducted with different maximum epoch limits. Results are shown in Table 4. The optimal performance was observed with a maximum training epoch set to 3,000, which ensured sufficient learning while avoiding unnecessary resource consumption. Increasing the limit to 4,000 epochs resulted in minimal gains in training epochs and cumulative reward, indicating 3,000 epochs as a reasonable upper bound for this task.

**Table 4:** Effect of maximum training epochs on model convergence.

**Maximum training epochs**	**Actual average epochs**	**Convergence rate (%)**	**Average cumulative reward**	**Early stopping applied**
1,000	960	91.5	51.1	NO
2,000	1,480	96.2	55.8	Yes
3,000	1,760	98.4	57.8	Yes (Recommended)
4,000	1,810	98.6	57.7	Yes

### 4.5 Intervention simulation results analysis

To assess the effectiveness of the reinforcement learning model in mental health interventions, simulation experiments based on the learned policy were designed. Table 5 presents the changes in mental health indicators under three intervention strategies: random, fixed, and reinforcement learning optimized strategies.

**Table 5:** Comparison of mental health improvement under simulated intervention strategies.

**Intervention strategy**	**Average anxiety score reduction**	**Average depression score reduction**	**Average convergence epochs**	**Average cumulative reward**
Random intervention strategy	1.2	1.0	–	–
Fixed intervention strategy	1.8	1.5	–	–
Reinforcement learning strategy	2.8	3.3	1,750	57.8

As presented in Table 5, the intervention strategy optimized through reinforcement learning significantly outperforms both random and fixed strategies, yielding ~55 and 120% greater reductions in anxiety and depression scores, respectively. By dynamically adjusting intervention measures and tailoring strategies to the heterogeneous psychological states of students, the model enhances the precision and effectiveness of the interventions. Furthermore, the model converges after an average of 1,750 training epochs, demonstrating robust training stability and effective learning performance. The average cumulative reward of 57.8 reflects an overall improvement in intervention outcomes, indicating that the reinforcement learning strategy effectively guides decision-making to facilitate sustained mental health improvements. These simulation results underscore the considerable potential of intelligent, reinforcement learning-based intervention strategies to support mental health management through more precise and personalized care. Nevertheless, given the simulated nature of this study, future research should incorporate clinical trial data to validate the model's practical efficacy and applicability in real-world settings.

### 4.6 Discussion

The results reveal that depression and anxiety scores among college students are generally elevated and display substantial individual variability. This observation was consistent with the findings of (Tadi et al. 2022), who identified college students as a high-risk population for mental health issues, characterized by complex and heterogeneous emotional disorders. Consequently, intervention strategies should move beyond a “one-size-fits-all” approach, instead tailoring interventions to individual psychological profiles and temporal changes to achieve more effective emotional regulation.

Simultaneously, data concerning social support and quality of life indicate that the majority of students report strong support networks and high levels of life satisfaction. These findings align with (Wang et al. 2023), who underscored the critical buffering role of social support in facilitating psychological adjustment among college students. However, a subset of students reported poor physical health and diminished psychological wellbeing, suggesting that interventions should adopt a holistic approach. Such an approach would address physical, emotional, social, and environmental domains to provide comprehensive psychological care.

In terms of technology use behaviors, the data show frequent smartphone and social media engagement among college students, with some subgroups using these platforms multiple times per hour. While such behaviors enhance information access and social connectivity, they may also contribute to increased psychological burden. (Bin and Sun 2022) demonstrated a significant association between excessive social media use and heightened symptoms of anxiety and depression, potentially mediated by sleep disturbances, attentional deficits, and negative social comparisons. Therefore, incorporating digital literacy education into intervention designs is imperative to promote healthy technology habits and mitigate adverse effects such as “information overload” and “social fatigue.”

Furthermore, sensitivity analyses of the reinforcement learning model's hyperparameters identified that a learning rate of 0.5, a discount factor of 0.8, and a maximum of 3,000 training epochs produced optimal convergence speed and intervention efficacy. This outcome validates the model's robustness under carefully tuned parameters and offers valuable guidance for the future deployment of similar psychological intervention frameworks. Most importantly, the significant reductions in anxiety and depression scores affirm the practical effectiveness of the model's behavior-driven intervention logic. In summary, this study provides empirical and mechanistic insights through comprehensive analysis of college students' mental health status, social support structures, new media usage patterns, and reinforcement learning model performance. These findings establish a data-driven foundation for the development of innovative, AI-enabled, precision-targeted psychological service pathways.

## 5 Conclusion

This study provides a systematic examination of the application of AI in personalized psychological interventions for university students within the context of new media technologies. Using large-scale datasets, it develops a reinforcement learning–based dynamic optimization framework that enables precise, individualized adjustment of intervention strategies based on students' mental health status. Simulation results show that AI-driven interventions significantly improve mental health outcomes, particularly in reducing symptoms of anxiety and depression. These interventions also account for important factors such as social support, quality of life, and new media usage patterns. Theoretically, the study integrates AI methodologies with established psychological intervention models, addressing the limitations of traditional static approaches by introducing a dynamic, data-driven, and personalized intervention paradigm. This innovation fills a critical gap in the digital transformation of mental health services within higher education. From a practical perspective, the study proposes viable technical solutions and strategic guidance to support the enhancement of university mental health services, underscoring considerable potential for broader implementation and scalability.

Nonetheless, several limitations warrant consideration. The dataset primarily represents U.S. university populations, and cultural and educational contextual differences may limit the generalizability of the findings. Future research should focus on validating and adapting the model across diverse cultural and regional populations to improve its applicability. Additionally, the real-world effectiveness and long-term impact of the AI-based intervention framework remain to be rigorously evaluated through clinical trials and longitudinal studies. Moving forward, expanding data inputs to include multimodal behavioral measures and improving the model's real-time adaptability and responsiveness are recommended. Furthermore, fostering interdisciplinary collaboration will be essential to deepen the integration of AI technologies with psychological practice, thereby advancing university mental health services toward greater intelligence, personalization, and precision. Therefore, this study advocates for increased scholarly attention and investment in AI-driven mental health interventions, promoting the transition from theoretical development to practical application. Such efforts are critical to delivering actionable solutions and academic support for improving college students' psychological wellbeing and addressing the mental health challenges posed by the digital era.

## Data Availability

The original contributions presented in the study are included in the article/supplementary material, further inquiries can be directed to the corresponding author.
